# Risk factors and nomogram for cognitive impairment after stereotactic drainage of spontaneous intracerebral hemorrhage

**DOI:** 10.3389/fneur.2026.1771378

**Published:** 2026-03-25

**Authors:** Min Gong, Xiaohong Fu, Yuanjun Xin, Hang Li, Shaofu Zhang

**Affiliations:** Department of Cerebrovascular Diseases, The First People’s Hospital of Zunyi, The Third Affiliated Hospital of Zunyi Medical University, Zunyi, Guizhou, China

**Keywords:** cognitive impairment, nomogram, post-stroke cognitive impairment, risk factors, spontaneous intracerebral hemorrhage, stereotactic puncture and drainage

## Abstract

**Objective:**

To investigate the risk factors for cognitive impairment after stereotactic puncture and drainage for spontaneous intracerebral hemorrhage (SICH) and to establish a nomogram prediction model.

**Methods:**

A retrospective analysis was conducted on 143 patients who underwent stereotactic puncture and drainage for SICH between June 2022 and June 2024. Patients were divided into cognitive impairment group (*n* = 57) and non-cognitive impairment group (*n* = 86) based on Montreal Cognitive Assessment at 3 months postoperatively. Univariate and multivariate logistic regression analyses were performed to identify independent risk factors, and a nomogram was constructed.

**Results:**

The incidence of cognitive impairment at 3 months was 39.86% (57/143). Multivariate logistic regression identified five independent risk factors: age (OR = 1.145, 95% CI: 1.089–1.205, *p* < 0.001), preoperative GCS score (OR = 0.512, 95% CI: 0.387–0.677, *p* < 0.001), hematoma volume (OR = 1.067, 95% CI: 1.028–1.108, *p* = 0.001), basal ganglia hemorrhage (OR = 5.567, 95% CI: 2.748–12.334, *p* < 0.001), and postoperative intracranial pressure (OR = 1.315, 95% CI: 1.168–1.481, *p* < 0.001). A nomogram integrating these five predictors was developed.

**Conclusion:**

Advanced age, lower preoperative GCS score, larger hematoma volume, basal ganglia hemorrhage, and elevated postoperative intracranial pressure are independent risk factors for cognitive impairment at 3 months following stereotactic drainage for SICH. The nomogram may assist in early identification of high-risk patients, though external validation is warranted.

## 1 Introduction

Spontaneous intracerebral hemorrhage (SICH) represents approximately 10–15% of all strokes and remains one of the most devastating cerebrovascular events, with significant long-term disability among survivors (1). Minimally invasive surgical techniques, particularly stereotactic puncture and drainage, have emerged as promising alternatives to traditional craniotomy, offering reduced surgical trauma while achieving effective hematoma evacuation (2). Evidence from pivotal multicenter trials such as MISTIE III and ENRICH has established that stereotactic aspiration with thrombolytic therapy can safely reduce perihematomal edema and is associated with improved functional outcomes (3, 4).

While these surgical advances have substantially improved survival, cognitive impairment remains a major concern among ICH survivors that profoundly impacts quality of life and functional independence. Post-stroke cognitive impairment encompasses a spectrum of cognitive deficits including impairments in memory, attention, executive function, and processing speed, and can occur regardless of treatment modality (5). The reported incidence of cognitive impairment following ICH varies widely across the literature, with individual studies in the systematic review by Kazim et al. (6) reporting rates ranging from 19 to 87.5%, a variation attributed to differences in diagnostic criteria, assessment tools, and patient populations. Their meta-analytic work synthesizing this data found a pooled prevalence of 46% (95% CI: 35.9–55.9%). Emerging evidence suggests that cognitive outcomes after intracerebral hemorrhage are influenced by multiple interconnected factors including age, hemorrhage characteristics, and perioperative management (7, 8).

Identifying risk factors for cognitive impairment in surgically treated ICH patients carries important clinical implications. Recognition of high-risk patients enables implementation of targeted monitoring and neuroprotective interventions, while robust risk assessment facilitates informed consent and helps establish realistic postoperative expectations with patients and families. Critically, modifiable risk factors represent actionable targets for therapeutic intervention to optimize cognitive outcomes. Recent studies have identified advanced age, hemorrhage volume and location, initial neurological severity, and postoperative intracranial pressure (ICP) as potential predictors of cognitive decline (9–11).

Despite growing recognition of the clinical significance of cognitive impairment following ICH, validated prediction models specifically designed for patients undergoing stereotactic drainage remain scarce. Existing nomograms demonstrate limited applicability to neurosurgical populations, particularly this unique population of ICH patients undergoing surgical treatment (12, 13). This study aims to identify independent risk factors for cognitive impairment at 3 months in patients who underwent stereotactic puncture and drainage for SICH, and to develop a clinically applicable nomogram based on readily available clinical variables. Such a tool could facilitate individualized risk assessment, guide postoperative rehabilitation planning, and ultimately improve patient outcomes through targeted interventions.

## 2 Materials and methods

### 2.1 Study population

**Figure 1:**
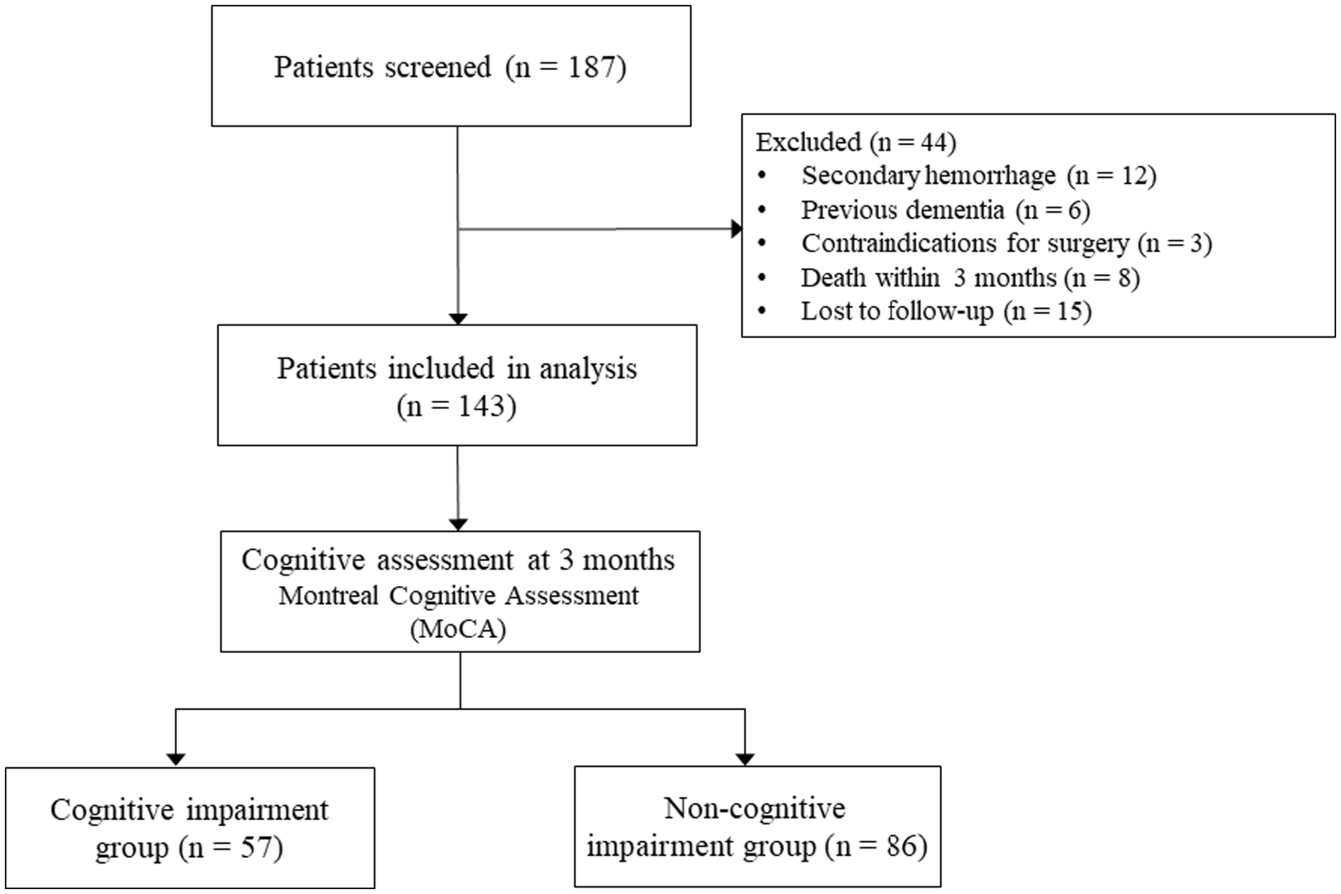
Study flowchart.

This retrospective study was conducted at our hospital between June 2022 and June 2024. During this period, 187 consecutive patients underwent stereotactic puncture and drainage for spontaneous intracerebral hemorrhage. Among the 187 screened patients, 44 were excluded: 12 had secondary hemorrhage, 6 had previous dementia, 3 had contraindications for surgery, 8 died within 3 months, and 15 were lost to follow-up. The final analytical cohort comprised 143 patients who met all inclusion criteria and completed 3-month cognitive assessment. This study was approved by the Ethics Committee of The First People’s Hospital of Zunyi [Approval No. Ethical Review (2022)-1-121, Date: May 22, 2022]. Informed consent was obtained verbally from all participants. The study flowchart is presented in Figure 1.

Inclusion criteria: (1) age ≥18 years; (2) SICH confirmed by computed tomography (CT); (3) hematoma volume ≥20 mL; (4) surgery within 24 h of symptom onset; (5) complete cognitive assessment at 3 months. Exclusion criteria: (1) secondary hemorrhage; (2) previous dementia; (3) contraindications for surgery; (4) death within 3 months; (5) loss to follow-up.

### 2.2 Surgical procedure

All procedures were performed using standardized stereotactic technique. Preoperative CT scans determined optimal trajectory. A drainage catheter was inserted under stereotactic guidance with gradual hematoma aspiration followed by urokinase irrigation.

### 2.3 Cognitive assessment and outcome definition

Cognitive function was assessed at 3 months postoperatively (91 ± 3 days after surgery, calculated from the date of operation) using the Beijing version of the Montreal Cognitive Assessment (MoCA-Beijing) (14). The MoCA is a 30-point screening tool that evaluates multiple cognitive domains including visuospatial/executive function, naming, attention, language, abstraction, delayed recall, and orientation, and has been validated for detecting cognitive impairment in stroke populations (15, 16). All assessments were administered by trained research nurses with more than 1 year of experience in cognitive assessment. Assessments were conducted in a quiet, standardized examination room during outpatient follow-up visits. Standard education-level adjustment was applied according to the original MoCA guidelines, with one point added to the total score for individuals with ≤12 years of formal education (2). Cognitive impairment at 3 months was defined as an adjusted MoCA score <26. Patients meeting this criterion were classified into the cognitive impairment group (*n* = 57), and those with MoCA score ≥26 were classified into the non-cognitive impairment group (*n* = 86).

### 2.4 Data collection

Clinical data collected included: (1) demographics: age, gender, education level; (2) medical history: hypertension, smoking, alcohol consumption, diabetes, hyperlipidemia; (3) preoperative parameters: Glasgow Coma Scale (GCS) score, hematoma volume and location; (4) surgical parameters: anesthesia time, operative time; (5) postoperative parameters: ICP at 1 h, American Society of Anesthesiologists (ASA) classification, National Nosocomial Infections Surveillance (NNTS) grading, ventricular involvement; (6) hospital stay.

### 2.5 Statistical analysis

Continuous variables were tested for normality using the Shapiro–Wilk test. Normally distributed data were expressed as mean ± standard deviation and compared using independent *t*-tests, while non-normally distributed data were presented as median (interquartile range) and analyzed using Mann–Whitney *U* tests. Categorical variables were presented as frequencies and percentages and compared using chi-square tests or Fisher’s exact tests when expected cell frequencies were less than 5.

Variables with *p* < 0.05 in univariate analysis were entered into multivariate logistic regression using backward stepwise elimination (removal criterion: *p* > 0.10). Multicollinearity was assessed using variance inflation factor (VIF <3). Sample size adequacy was evaluated using events per variable (EPV) ratio. Model fit was evaluated using the Hosmer–Lemeshow goodness-of-fit test. Odds ratios with 95% confidence intervals were calculated.

A nomogram was constructed based on the final regression model using the rms package in R. Internal validation was performed using bootstrap resampling with 1,000 repetitions. Optimism-corrected performance metrics were calculated by subtracting the average optimism (difference between bootstrap performance and test performance) from the apparent performance. Model discrimination was assessed by the area under the receiver operating characteristic curve (AUC) with 95% confidence interval. Calibration was evaluated using calibration plots and quantitative metrics including calibration slope, calibration intercept, mean absolute error, and Brier score. The optimal cutoff was determined using the Youden index (calculated as sensitivity + specificity − 1). Calibration was evaluated using calibration plots and mean absolute error. Clinical utility was assessed using decision curve analysis.

Missing data were minimal (<2%) and occurred only in variables not retained in the final model (smoking history, *n* = 2; hospital stay, *n* = 1). All five variables included in the final prediction model had complete data for all 143 patients. Complete case analysis was therefore performed without imputation. Analyses were performed using SPSS 26.0 (IBM Corp.) and R 4.2.0 (R Foundation) with packages including “rms,” “pROC,” “rmda,” and “ggplot2.” Two-sided *p* < 0.05 was considered statistically significant.

## 3 Results

### 3.1 Baseline characteristics and univariate analysis

A total of 143 patients were included, with 57 (39.86%) meeting the criteria for cognitive impairment at 3 months. Significant differences were observed between groups in age, male gender, education level, preoperative GCS score, hematoma volume, hematoma location, operative time, and postoperative ICP (see Table 1).

**Table 1:** Comparison of clinical characteristics between cognitive impairment and non-cognitive impairment groups.

Characteristic	Cognitive impairment group (*n* = 57)	Non-cognitive impairment group (*n* = 86)	Statistic	*p*-value
Age (years)	69.33 ± 11.15	50.42 ± 12.15	*t* = 9.246	<0.001
Male, *n* (%)	38 (66.67)	42 (48.84)	*χ*^2^ = 4.373	0.037
Education level >9 years, *n* (%)	22 (38.60)	54 (62.79)	*χ*^2^ = 10.234	0.006
Hypertension, *n* (%)	34 (59.65)	47 (54.65)	*χ*^2^ = 0.342	0.559
Smoking, *n* (%)	33 (57.89)	50 (58.14)	*χ*^2^ = 0.001	0.978
Alcohol consumption, *n* (%)	32 (56.14)	44 (51.16)	*χ*^2^ = 0.337	0.562
Diabetes mellitus, *n* (%)	30 (52.63)	47 (54.65)	*χ*^2^ = 0.056	0.813
Hyperlipidemia, *n* (%)	34 (59.65)	50 (58.14)	*χ*^2^ = 0.032	0.858
Preoperative GCS score	6 (5–7)	9 (7–11)	*Z* = −6.758	<0.001
Hematoma volume (mL)	45.82 ± 11.35	35.67 ± 9.84	*t* = 5.592	<0.001
Hematoma location, *n* (%)			*χ*^2^ = 45.123	<0.001
Basal ganglia	47 (82.46)	28 (32.56)		
Lobar	6 (10.53)	31 (36.05)		
Cerebellum	3 (5.26)	17 (19.77)		
Brainstem	1 (1.75)	10 (11.63)		
Anesthesia time (min)	70.64 ± 8.24	70.06 ± 8.12	*t* = 0.419	0.676
Operative time (min)	56.64 ± 5.25	52.55 ± 5.29	*t* = 4.551	<0.001
Postoperative ICP at 1 h (cmH₂O)	26.71 ± 3.83	22.26 ± 3.82	*t* = 6.772	<0.001
ASA P1/P2, *n* (%)	49/8 (85.96/14.04)	69/17 (80.23/19.77)	*χ*^2^ = 0.823	0.364
NNTS grade 0/2, *n* (%)	51/6 (89.47/10.53)	72/14 (83.72/16.28)	*χ*^2^ = 1.007	0.316
Ventricular involvement, *n* (%)	48 (84.21)	75 (87.21)	*χ*^2^ = 0.269	0.604
Hospital stay (days)	23.40 ± 14.31	20.13 ± 9.28	*t* = 1.639	0.104

Continuous variables are presented as mean ± standard deviation or median (interquartile range) as appropriate. GCS, Glasgow Coma Scale; ICP, intracranial pressure; IQR, interquartile range; ASA, American Society of Anesthesiologists; NNTS, National Nosocomial Infections Surveillance.

### 3.2 Multivariate analysis of risk factors

Eight variables with *p* < 0.05 in univariate analysis were entered into multivariate logistic regression: age, gender, education level, preoperative GCS score, hematoma volume, hematoma location, operative time, and postoperative ICP. Multicollinearity diagnostics revealed no significant collinearity among the variables (Table 2). All variance inflation factors (VIF) were below 2.0, well within the acceptable threshold of 3.0, indicating that the predictors were sufficiently independent for reliable regression analysis.

**Table 2:** Multicollinearity diagnostics for independent variables.

Variable	VIF
Age	1.342
Male gender	1.156
Education level	1.789
Preoperative GCS score	1.523
Hematoma volume	1.267
Basal ganglia hemorrhage	1.234
Operative time	1.189
Postoperative ICP at 1 h	1.298

VIF, variance inflation factor; GCS, Glasgow Coma Scale; ICP, intracranial pressure. VIF <3 indicates acceptable multicollinearity.

After backward stepwise selection, five independent risk factors were identified (Table 3): age, preoperative GCS score, hematoma volume, basal ganglia hemorrhage, and postoperative ICP. During the stepwise selection process, gender (*p* = 0.338), education level (*p* = 0.284), and operative time (*p* = 0.226) were removed from the final model due to lack of statistical significance after adjustment for other covariates. Sample size adequacy was confirmed with an events per variable ratio of 11.4 (57 events/5 predictors). The Hosmer–Lemeshow goodness-of-fit test showed no evidence of poor fit (*χ*^2^ = 6.234, df = 8, *p* = 0.621), indicating good calibration of the model.

**Table 3:** Multivariate logistic regression analysis of risk factors for cognitive impairment.

Risk factor	*β*	SE	OR	95% CI	*p*-value
Intercept	−13.208	2.154	—	—	<0.001
Age (per year)	0.135	0.026	1.145	1.089–1.205	<0.001
Preoperative GCS score	−0.669	0.143	0.512	0.387–0.677	<0.001
Hematoma volume (per mL)	0.065	0.019	1.067	1.028–1.108	0.001
Basal ganglia hemorrhage	1.717	0.384	5.567	2.748–12.334	<0.001
Postoperative ICP at 1 h (per cmH₂O)	0.274	0.061	1.315	1.168–1.481	<0.001

*β*, regression coefficient; SE, standard error; OR, odds ratio; CI, confidence interval; GCS, Glasgow Coma Scale; ICP, intracranial pressure. Basal ganglia hemorrhage is coded as 1 for basal ganglia location and 0 for other locations (lobar, cerebellum, or brainstem). OR >1 indicates increased risk of cognitive impairment; OR <1 indicates decreased risk (protective factor).

To facilitate external validation and reproducibility in accordance with TRIPOD guidelines, the full logistic regression equation is provided: Logit(*p*) = −13.208 + 0.135 × Age − 0.669 × GCS + 0.065 × Volume + 1.717 × Basal ganglia + 0.274 × ICP. Where *p* represents the predicted probability of cognitive impairment at 3 months, Age is measured in years, GCS is the preoperative Glasgow Coma Scale score, Volume is the hematoma volume in mL, Basal ganglia is coded as 1 for basal ganglia hemorrhage and 0 for other locations, and ICP is the postoperative intracranial pressure at 1 h in cmH₂O. The predicted probability can be calculated as: *p* = 1/(1 + e^−Logit(*p*)^).

### 3.3 Nomogram development and internal validation

**Figure 2:**
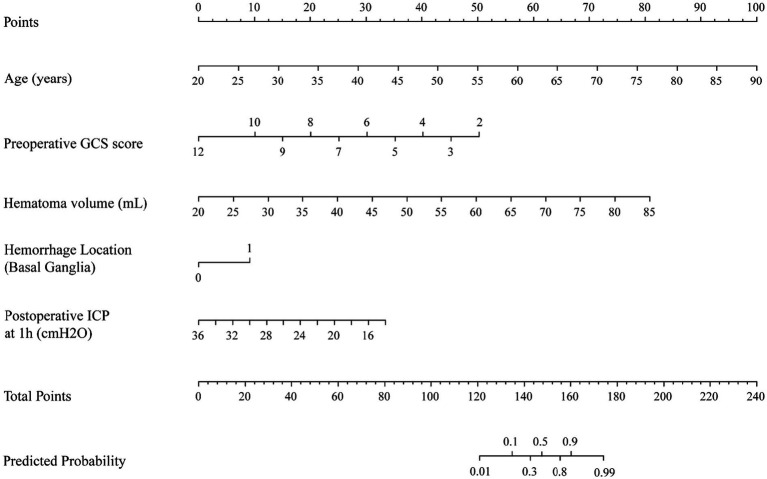
Nomogram for predicting cognitive impairment at 3 months following stereotactic puncture and drainage for spontaneous intracerebral hemorrhage. To use the nomogram: locate patient values on each axis, draw a line upward to the points axis to determine points for each variable, sum all points to get total points, then draw a line downward from total points to obtain the predicted probability of cognitive impairment.

**Figure 3:**
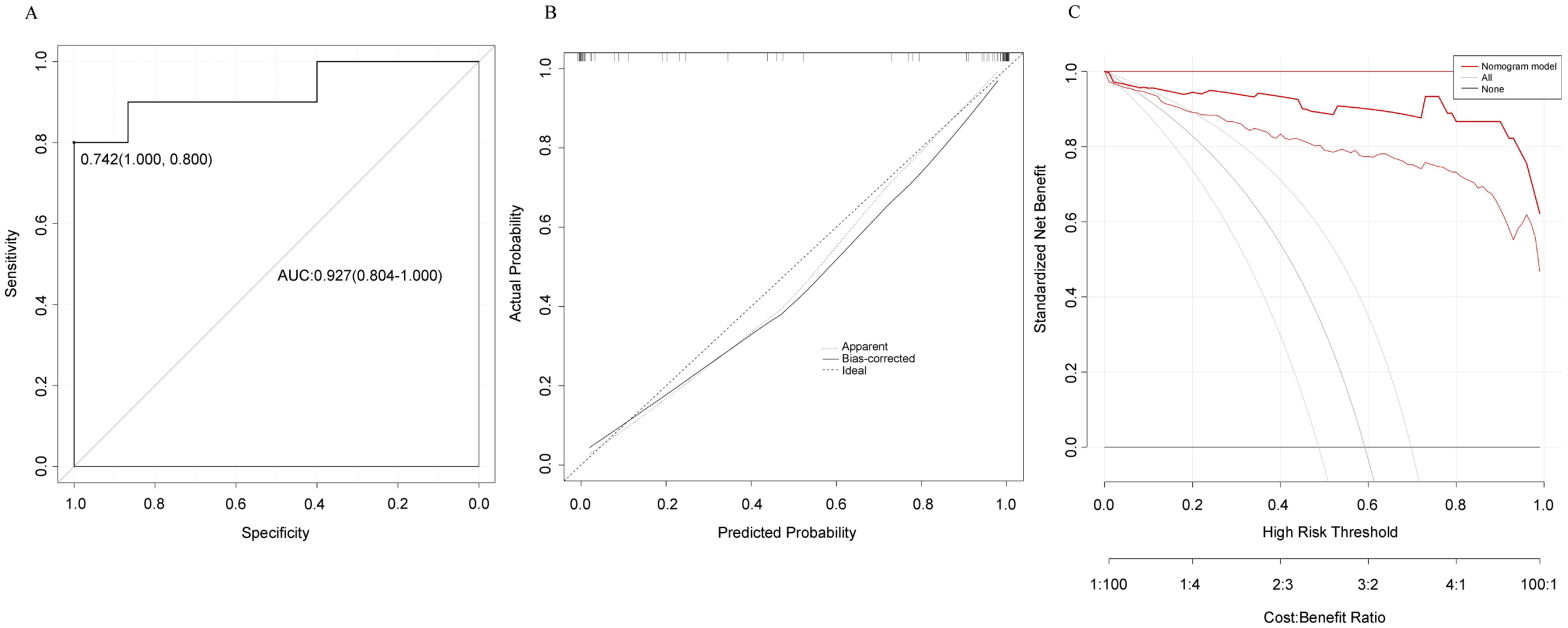
Model performance evaluation. **(A)** ROC curve showing discriminative ability (AUC = 0.927, 95% CI: 0.804–1.000). Optimal cutoff = 0.40 with sensitivity 74.2% and specificity 80.0%. **(B)** Calibration curve demonstrating agreement between predicted and observed probabilities. Mean absolute error = 0.038. **(C)** Decision curve analysis showing net benefit across threshold probabilities 0–80%, indicating clinical utility of the model. AUC, area under the curve; ROC, receiver operating characteristic.

Based on the five independent risk factors, a nomogram prediction model was constructed (Figure 2). Each predictor was assigned points based on its regression coefficient, and total points were calculated to predict the probability of cognitive impairment. The model demonstrated good discriminative ability with an apparent AUC of 0.927 (95% CI: 0.804–1.000) (Figure 3A). Bootstrap internal validation with 1,000 repetitions was performed to assess optimism and model stability. The estimated optimism was 0.021, yielding an optimism-corrected AUC of 0.906, indicating that the model retains good discrimination after accounting for potential overfitting. At the optimal cutoff value of 0.40, the model achieved sensitivity of 74.2% and specificity of 80.0%. Youden index = sensitivity + specificity – 1 = 0.742 + 0.800 − 1 = 0.542.

The calibration curve showed good agreement between predicted and observed probabilities (Figure 3B). The calibration slope was 0.912 (ideal = 1.0) and calibration intercept was −0.036 (ideal = 0), indicating minimal overfitting and good calibration-in-the-large. The mean absolute error was 0.038 and Brier score was 0.128, collectively demonstrating adequate predictive accuracy. Decision curve analysis demonstrated that the nomogram provided clinical net benefit across threshold probabilities ranging from 0 to 80% (Figure 3C), supporting its clinical utility for decision-making.

## 4 Discussion

This study identified five independent risk factors for cognitive impairment at 3 months following stereotactic puncture and drainage of spontaneous intracerebral hemorrhage: advanced age, lower preoperative Glasgow Coma Scale score, larger hematoma volume, basal ganglia hemorrhage location, and elevated postoperative ICP. The nomogram prediction model demonstrated good discriminative ability and adequate calibration, suggesting potential utility for early identification of high-risk patients in clinical practice.

The observed incidence of cognitive impairment (39.86%) in our cohort is consistent with the reported prevalence of cognitive impairment following intracerebral hemorrhage. A recent systematic review and meta-analysis by Kazim et al. (6) documented cognitive impairment prevalence of 46% (95% CI: 35.9–55.9%) following intracerebral hemorrhage, with substantial heterogeneity attributed to variations in assessment timing, diagnostic criteria, and patient populations. Our rate of 39.86% at 3 months aligns closely with this pooled estimate and reflects assessment at a critical transitional period when acute recovery effects are resolving while structural and functional reorganization continues.

Importantly, the five independent predictors retained in our final model—age, preoperative GCS score, hematoma volume, basal ganglia location, and postoperative ICP—are all markers of hemorrhage severity and secondary brain injury rather than surgery- or anesthesia-specific factors. Notably, perioperative variables including anesthesia time and operative time were not retained in multivariable analysis despite showing univariate associations. This pattern suggests that the observed cognitive impairment is primarily attributable to the intracerebral hemorrhage itself and its sequelae, rather than representing a distinct surgery- or anesthesia-induced phenomenon. Therefore, the cognitive outcomes in our cohort may be more appropriately conceptualized within the framework of post-stroke cognitive impairment rather than traditional postoperative cognitive dysfunction as described in non-neurological surgical populations (17).

Age emerged as a prominent risk factor, with each additional year conferring a 14.5% increase in cognitive impairment odds. This association reflects well-established neurobiological mechanisms including age-related reductions in cerebral metabolic reserve, diminished neuroplasticity, and increased vulnerability to perioperative neuroinflammation (18). Elderly patients demonstrate compromised blood–brain barrier integrity and heightened susceptibility to secondary brain injury cascades following intracerebral hemorrhage (19). Recent diffusion tensor imaging studies have demonstrated that white matter injury following hemorrhage is strongly associated with cognitive impairment (20), with age-related white matter degeneration amplifying this vulnerability in elderly patients (21), potentially explaining the heightened cognitive vulnerability observed in our cohort. These findings underscore the necessity for age-stratified risk assessment and consideration of neuroprotective strategies in elderly surgical candidates.

Preoperative neurological severity, quantified by GCS score, demonstrated strong inverse correlation with cognitive impairment risk. Lower admission GCS reflects greater primary injury burden, extensive perihematomal edema, and potential mass effect with secondary tissue compression (22). Patients presenting with profound neurological impairment face cumulative physiological insults including prolonged hypoperfusion, inflammatory mediator release, and disrupted cerebral autoregulation, all contributing to neuronal injury extending beyond the hemorrhage epicenter (23). The protective OR of 0.512 per GCS point emphasizes the critical importance of initial neurological status in determining cognitive trajectories. This observation aligns with data from the CLEAR III and MISTIE III trials, where baseline neurological function consistently predicted long-term functional outcomes despite successful hematoma evacuation (3, 4).

Hematoma volume demonstrated independent association with cognitive impairment, with each milliliter increase conferring 6.7% higher odds. Volumetric expansion exerts direct destructive effects through tissue disruption while simultaneously triggering secondary injury mechanisms including inflammatory responses, oxidative stress, and excitotoxicity (24). Larger hemorrhages generate extensive perihematomal metabolic derangements detectable on advanced neuroimaging, correlating with cognitive domain-specific impairments in executive function and processing speed (9, 25). Contemporary evidence from minimally invasive surgery trials suggests that effective volume reduction correlates with improved functional outcomes, though the cognitive benefits require further delineation through dedicated neuropsychological assessment protocols (26, 27). The recent ENRICH trial demonstrated that early surgical intervention for lobar ICH improved functional outcomes, suggesting potential cognitive benefits from timely hematoma evacuation that warrant further investigation (28). Our findings support aggressive yet judicious evacuation strategies while recognizing that volume alone incompletely captures the complexity of hemorrhage-induced cognitive injury.

Basal ganglia hemorrhage location exhibited particularly strong association with cognitive impairment, conferring 5.6-fold increased odds compared to other locations. We dichotomized hemorrhage location as basal ganglia versus non-basal ganglia for multivariable analysis based on both pathophysiological rationale and practical considerations. The relatively small sample sizes for brainstem (*n* = 11) and cerebellar (*n* = 20) hemorrhages precluded reliable location-specific subgroup analysis. Moreover, prior literature consistently identifies basal ganglia involvement as a key determinant of cognitive outcomes following ICH (29). This anatomical predilection likely reflects disruption of critical frontostriatal circuits integral to executive function, working memory, and cognitive flexibility (30). The basal ganglia serve as key relay stations within networks connecting prefrontal cortex, thalamus, and striatum, with hemorrhagic disruption precipitating widespread network dysfunction extending beyond focal structural damage (31, 32). Hemorrhagic disruption of these structures precipitates widespread network dysfunction extending beyond focal structural damage. Recent connectome analyses utilizing resting-state functional MRI have demonstrated that basal ganglia hemorrhages induce profound alterations in large-scale brain network topology, with reduced integration and increased segregation patterns persisting months after ictus (33, 34). Additionally, basal ganglia proximity to internal capsule and thalamic nuclei increases risk of secondary degeneration through wallerian degeneration and diaschisis phenomena, further compounding cognitive vulnerability (35).

Postoperative ICP elevation emerged as potentially a modifiable risk factor, with each cmH₂O increase raising cognitive impairment odds by 31.5%. Elevated ICP compromises cerebral perfusion pressure, inducing global ischemia and exacerbating secondary injury cascades (36). Even transient ICP spikes during the acute postoperative period can trigger detrimental processes including blood–brain barrier disruption, astrocyte swelling, and neuronal apoptosis (37, 38). The delayed onset of cognitive impairment at 3 months suggests that early ICP perturbations initiate progressive neurodegenerative processes rather than purely acute effects. This temporal dissociation between perioperative ICP elevation and delayed cognitive manifestation aligns with emerging concepts of hemorrhage-induced chronic neurodegeneration mediated by persistent microglial activation and tau pathology (39, 40). These findings emphasize the critical importance of meticulous postoperative ICP management and suggest potential benefits from extended monitoring protocols in high-risk patients.

The nomogram model exhibited adequate calibration and potential clinical utility, with decision curve analysis demonstrating net benefit across threshold probabilities ranging from 0 to 80%. This practical tool enables individualized risk stratification at the bedside by integrating readily available clinical parameters without requiring advanced imaging or biomarker assessment. Although internal validation with optimism correction demonstrated that the model retains good predictive performance, these estimates remain derived from a single-center retrospective cohort, and independent external validation is necessary to confirm generalizability. Potential clinical applications include enhanced informed consent processes, selective allocation of intensive monitoring resources, targeted implementation of multimodal neuroprotective bundles including optimal blood pressure management and glycemic control, and early identification of candidates for cognitive rehabilitation interventions.

Several limitations of this study warrant careful consideration. The retrospective single-center design may limit generalizability and introduces potential selection bias. Notably, 23 patients (12.3%) were excluded from analysis due to death within 3 months (*n* = 8) or loss to follow-up (*n* = 15). These excluded patients may have had more severe hemorrhages or worse baseline status, potentially leading to underestimation of the true cognitive impairment incidence and overestimation of model performance. We were unable to perform a formal comparison of baseline characteristics between included and excluded patients due to incomplete data availability for those lost to follow-up, and future prospective studies should implement strategies to minimize attrition and conduct sensitivity analyses to address this potential survivor bias.

Our modeling approach using univariable screening followed by backward stepwise selection is widely adopted in clinical prediction research for its interpretability and ease of clinical implementation; however, this approach has recognized limitations, including potential inflation of effect estimates, particularly in moderate sample sizes. To address these concerns, we implemented several methodological safeguards: we confirmed an adequate events per variable ratio (EPV = 11.4, exceeding the recommended minimum of 10), assessed multicollinearity among predictors (all VIF <2.0), and performed bootstrap internal validation with 1,000 repetitions to estimate and correct for optimism. The resulting optimism-corrected AUC of 0.906 and calibration slope of 0.912 suggest that the model retains adequate predictive performance after accounting for potential overfitting. Nevertheless, the relatively modest sample size (*n* = 143) precluded independent external validation, and these performance estimates remain derived from internal validation. Future multicenter studies with larger sample sizes should consider penalized regression methods (such as LASSO) to further assess model stability, and independent external validation in different populations remains essential before clinical implementation.

Assessment at a single three-month time point precludes characterization of cognitive trajectory evolution. Some patients may show delayed recovery beyond 3 months, while others may experience progressive decline; our cross-sectional design cannot distinguish these trajectories. Future prospective studies should incorporate serial assessments to capture recovery patterns and identify predictors of persistent versus transient dysfunction. Furthermore, while MoCA provides validated global cognitive screening, it may have ceiling effects in patients with milder impairment and does not provide detailed assessment of specific cognitive domains. Comprehensive neuropsychological batteries would enable domain-specific profiling essential for mechanistic insights and targeted intervention development. The model also does not incorporate advanced neuroimaging biomarkers such as cerebral microbleeds, white matter hyperintensities, or structural connectivity metrics, nor inflammatory or genetic markers that have been associated with post-stroke cognitive outcomes—representing opportunities for model refinement in future iterations.

Despite these limitations, our findings carry important implications for clinical practice. Among the identified risk factors, age, GCS score, hematoma volume, and location are non-modifiable at the time of clinical decision-making; however, they can inform prognostication, patient counseling, and rehabilitation planning. Postoperative ICP management represents a potentially modifiable target, and standardized protocols for ICP control may improve cognitive outcomes, although this hypothesis requires prospective validation. Early identification of high-risk patients using this nomogram could facilitate timely referral for cognitive rehabilitation, implementation of neuroprotective strategies, and allocation of intensive monitoring resources. Future research should investigate whether targeted interventions in high-risk patients—including cognitive rehabilitation, pharmacological neuroprotection, or neuromodulation approaches—can modify cognitive trajectories. Multicenter prospective studies with standardized assessment protocols, longer follow-up periods, and external validation cohorts are needed to establish the clinical utility of this prediction model.

## 5 Conclusion

In conclusion, this study identifies five independent risk factors for cognitive impairment at 3 months following stereotactic drainage for spontaneous intracerebral hemorrhage and develops a nomogram prediction model. Internal validation demonstrated good discriminative ability and adequate calibration, suggesting potential utility for early identification of high-risk patients. However, external validation is warranted before clinical implementation. These findings underscore the multifactorial nature of post-hemorrhage cognitive outcomes and highlight opportunities for targeted interventions to optimize cognitive recovery in this vulnerable population.

## Data Availability

The original contributions presented in the study are included in the article/supplementary material, further inquiries can be directed to the corresponding author.
